# Triple-negative vimentin-positive heterogeneous feline mammary carcinomas as a potential comparative model for breast cancer

**DOI:** 10.1186/s12917-014-0185-8

**Published:** 2014-09-25

**Authors:** Diego Caliari, Valentina Zappulli, Roberta Rasotto, Barbara Cardazzo, Federica Frassineti, Michael H Goldschmidt, Massimo Castagnaro

**Affiliations:** Department Comparative Biomedicine and Food Science, University of Padua, Padua, Italy; Animal Health Trust, Newmarket, UK; Department of Pathobiology, School of Veterinary Medicine, University of Pennsylvania, Philadelphia, USA

**Keywords:** Mammary tumor, Feline, Human breast cancer, Vimentin, Markers, Triple-negative

## Abstract

**Background:**

Human breast cancer is a heterogeneous disease classified by molecular subtyping into luminal A, luminal B, HER2-overexpressing, basal-like, claudin-low and normal-breast like. The routinely applied and standardized immunohistochemical-based surrogates of this classification group together the last three entities as triple-negative breast cancer (TNBCs) that show the most diverse and complex heterogeneity and represent a therapeutic challenge.

In the present work 156 feline mammary lesions consisting of feline mammary carcinomas (FMCs), benign neoplasms, and hyperplastic/dysplastic tissues were evaluated histologically and by immunohistochemistry for expression of basal and luminal cytokeratins (CK), vimentin, alpha-smooth muscle actin, calponin, estrogen receptor (ER) alpha (a), and progesterone receptor (PR). Thirty-seven FMCs with 27 matched non-neoplastic controls were also investigated for gene expression of *ERa*, *ER beta*, *PR*, and *HER2*.

**Results:**

A large group of hormone receptors (HRs)-negative aggressive carcinomas - that did not overexpress HER2 - could be distinguished from the less aggressive (10.8%) and benign (8%) HRs + tumors, that showed bilineage (luminal and myoepithelial) differentiation. Immunohistochemical evaluations of cytoplasmic filaments indicated that HRs- FMCs are vimentin+, CK14+, and CK5_6+ carcinomas that may resemble the TNBCs (basal like/claudin low) described in women. The identification of luminal and myoepithelial progenitors within the mammary ductal system suggested potential cells/sites of origin of these tumors. A diffuse and never previously described CKs/vimentin luminal cell co-expression was detected in the non-neoplastic ducts, indicating a potential bilineage progenitor.

**Conclusions:**

These results indicate and potentially explain the high incidence of triple-negative, vimentin + aggressive tumors in cats that may used to elucidate some of the challenging features of TNBCs in women.

## Background

Human breast cancer (HBC) is a heterogeneous disease that still leads to more than 120.000 expected deaths per year [1]. One of the explanations for the high mortality rate is the complexity of the cellular components of the normal breast and the associated molecular mutations. By molecular analyses several distinct “intrinsic” HBC subtypes have been identified (*i.e*. luminal A, luminal B, HER2-overexpressing, basal-like, claudin-low, and normal-breast like) [2-4].

Because of the complexity and costs of gene expression profiling, immunohistochemical (IHC) surrogates of the molecular subtypes are routinely assessed [4,5]. Guidelines for IHC markers (*i.e*. ER, PR, HER2, Ki67) evaluation are continuously under discussion to stratify breast cancer patients in a clinical context for prognostic and treatment selection purposes [6-8]. When applying both clinical and IHC analysis to HBC a group of “triple negative” cancers (TNBCs) (lacking ER, PR, and HER2) is identified [9]. TNBCs constitute 10-20% of all HBCs, are frequently high-grade aggressive invasive ductal carcinomas, lack a specific targeted therapy, and are a heterogeneous group of breast tumors [10]. Approximately 70-75% of TNBCs share similarities with the basal-like breast cancer (BLBC) molecular subtype; therefore they have been considered erroneously as synonyms [9]. Histologically, the majority of TNBCs are invasive ductal carcinomas but medullary, metaplastic, and adenoid cystic histological subtypes share the triple negativity despite a more favorable prognosis [11]. Using transcriptome analysis distinct classes of TNBCs have been recognized: the BLBCs, the normal breast-like HBCs, and the newly identified claudin-low subtype [12,13], but further efforts dissected even more distinct TNBCs signatures [10].

At present, there is no standardization of IHC markers able to identify the TNBCs classes. BLBCs show expression of basal cytokeratins (CK5/6 and/or CK17 and/or CK14), epidermal growth factor receptor (EGFR), C-kit (CD117), and a high frequency (80%) of p53 mutation [12]. In contrast, claudin-low tumors show epithelial-to-mesenchymal transition (EMT) features, immune system responses, and stem cell-associated biological markers [13]. Many researchers have speculated that the genomically defined HBC subtypes may represent transformation of stem cells with arrest at specific stages of development or, alternatively, direct transformation of various mature cell types [3,5,14]. Data suggest that invasive HBCs may be placed on a normal mammary differentiation hierarchy and in this hypothetical view of developmental origin the claudin-low class would be considered as the most primitive subtype [3].

In the feline species, mammary cancer is a leading cause of death and the most common tumor in female cats (queens). Feline mammary tumors (FMTs) are frequently high-grade invasive carcinomas (80-90%) that lack a well-differentiated tumor-suppressor myoepithelial component which is much more common in canine mammary tumors [15,16]. Although there are no robust and standardized results for hormone receptors (HRs) and HER2 positivity, feline mammary carcinomas (FMCs) often lack significant levels of ERa and PR [17-23]. In addition, FMCs have been associated with decreased expression of adhesion molecules [24] and high expression of basal cytokeratins and vimentin [25], and a “basal-like” subtype was identified by IHC-analyses [17].

Further demonstration of these interesting similarities between FMCs and TNBCs might offer relevant information in veterinary medicine and might support FMC as a useful spontaneous model for pathogenetic mechanisms and therapeutic approaches [17,18,26], overcoming some of the limitations of HBC cell-line based studies and mouse modeling [27-30].

The aim of this study was to determine the immunohistochemical and molecular features of non-neoplastic mammary gland tissue and FMTs in term of HRs, HER2, and cytoplasmic filaments expression and to provide additional information on the possible origin of HRs- vimentin + FMTs that may be useful for further analyses of HBC.

## Methods

### Samples collection and follow-up data

The present IHC and molecular study was conducted on a population of 81 queens with mammary gland lesions. For IHC analysis paraffin-embedded samples, submitted to the Diagnostic Service of Veterinary Anatomical Pathology (University of Padua, Italy) routinely collected during surgery and processed as previously described [22], were used. Distant metastases were not reported at the time of diagnosis. In addition, for 37/81 cases a portion (approx. 4 × 4 × 6 mm) of enlarged mammary tissue was collected at the time of surgery and was stored in RNA Later (Ambion, Austin, TX) (−80°C). Twenty-seven samples of adjacent presumed normal mammary parenchyma (matched controls) were also collected immediately after surgery as the adjacent portion of the presumptive tumor with no further sampling for the subject and identically stored.

Data on the one-year post-surgical survival and the development of local relapses and distant metastases were available for 43 subjects. Twenty-two of 40 cats (55%) with malignant tumors were dead within the first year after surgery; 12/40 (30%) of the cats had visceral metastases and 17/40 (42.5%) had local recurrences.

### Histopathology

Histological evaluation of the paraffin-embedded samples from the 81 subjects was performed by two or more ECVP-certified pathologists. Morphological diagnoses were based on the WHO classification [15] and on the recent literature [16,31] that describes new mammary tumor subtypes that includes the new categories of comedocarcinoma, ductal adenoma/carcinoma, and intraductal papillary adenoma/carcinoma. The ductal and intraductal papillary (“ductal-associated”) tumors were confirmed by IHC as biphasic (see later). Criteria of malignancy were: significant nuclear/cellular pleomorphism, presence of random areas of necrosis, and mitotic index (MI). The MI was calculated as the total number of mitoses per 10 high power fields (40×, Olympus BX40) in the areas with the highest proliferative activity. A MI > 3 was used as cut-off for malignancy in borderline (benign *vs* malignant) lesions. Grading of malignant tumors was performed using the modified Elston and Ellis [32] system [33]. Peritumoral lymphatic invasion was also assessed. Before RNA extraction half of the RNA Later-preserved sample was embedded in paraffin for histological evaluation.

### IHC analysis

Four-micron-thick sections were cut, mounted on Superfrost®Plus microscope slides (Menzel GmbH, Braunschweig, Germany) and dried at 37°C for 30 minutes. IHC evaluations for cytokeratins (CK) CK5-6, CK14, CK8-18, panCK, calponin (CALP), vimentin (VIM), alpha-smooth muscle actin (aSMA), ERa, and PR were performed using an automated immunostainer (BenchMark XT®, Ventana Medical System Inc., Tucson, AZ). See Table 1 for specific primary antibodies and protocols. The incubation temperature for all the antibodies was 40°C and the *ultra*View Universal DAB detection Kit was applied (Ventana Medical System Inc., Tucson, AZ).

**Table 1:** **Antibodies and details of the protocols applied for the immunohistochemical examination**

**Markers**	**Antibodies** **(mouse anti** **-human) clone and producer**	Dilution*	**Unmasking**	Incubation time**
Cytokeratin 5/6	Clone D5/16 B4	1:100	CCR	24 min
	Dakocytomation		+ protease 2 min	
Cytokeratin 14	NCL –L-LL002	1:20	CCR	18 min
	Novocastra			
Cytokeratin 8/18	NCL-L-5D3	1:20	protease 8 min	24 min
	Dakocytomation			
Pancytokeratin	Clone AE1/AE3	1:100	CCR	16 min
	Dakocytomation			
Calponin	Clone Calp	1:200	CCR	12 min
	Dakocytomation			
Vimentin	Clone V9	1:100	CCR	18 min
	Dakocytomation			
α-smooth muscle actin	Clone 1A4	1:100	no	10 min
	Dakocytomation			
Estrogen receptor	NCL-ER-6 F11	1:40	CCS	14 min
	Novocastra			
Progesteron receptor	Clone: PR10A9	1:100	CCE	18 min
	Immunotech			

CCR, cell conditioning reduced: 30 minutes at 95°C; CCS, cell conditioning standard: 60 minutes at 95°C; CCE, cell conditioning extended: 90 minutes at 95°C.

*The primary antibody was diluted in the Antibody Diluent (Ventana Medical System Inc., Tucson, AZ).

**Incubation time for primary antibody; min, minutes.

Internal positive controls were the epidermis, adnexal epithelium, the non-neoplastic mammary glands (panCK, CK5_6, CK14, CK8_18), and vessels wall (CALP and aSMA). Sections of feline uterus were used as positive controls for ER and PR.

For all markers positivity was evaluated as the percentage of positive neoplastic cells counted in at least 10 random high-power fields (40×), avoiding necrotic areas and the immediately adjacent portions, for a total of 1,000 cells. Specifically, nuclear positivity was evaluated for ERa and PR, and samples were considered positive if >1% cells were stained, whereas cytoplasmic brown staining was considered as positive for CK5_6, CK14, CK8_18, panCK, CALP, VIM, and aSMA. For the latter markers the morphology and location of positive cells was determined, and a positive sample cut-off was not established.

Light microscopic evaluation of each marker was conducted in a blinded manner. Consensus was achieved with a third pathologist in discordant cases. Positivity was evaluated separately for all types of lesions (malignant, benign, hyperplastic) and the normal mammary tissue.

### RNA extraction and sequencing

Samples preserved in RNA Later (approx. half of the sample) including 37 tumoral and 27 normal glands, homogenized in Trizol Reagent (Invitrogen, Carlsbad CA) and total RNA isolation was completed following the manufacturer’s protocol. The extracted RNAs were treated with RQ1 RNAse-free DNAse (Promega, Madison, WI) and purified with a standard phenol–chloroform extraction.

One microgram of total RNA from each sample was reverse transcribed using a reverse transcriptase (Superscript II, Life technologies Grand Island, NY) and random hexamers to obtain first-strand cDNA. The cDNA was then used as a template for quantitative real-time PCR to evaluate the relative expression of *ERa*, *ER beta* (*b*), *PR*, *Erb*-*B2* genes in feline mammary lesions and matched controls. The real-time PCR MGB assay were designed using the Assay-by-design service (Life Technologies, Grand Island, NY) based on the coding sequences of the feline *ERa*, *ERb*, *PR*, *Erb*-*B2* as target genes and *b*-*Glucuronidase* (*b*-*Glu*) as the reference gene (Acc. Num. AY605260, HE608843, JX965384, AY702651, AF012423). An aliquot (2.5 μl) of diluted (1:50) cDNA template was amplified in a final volume of 10 μl, containing 5 μl of TaqMan® Universal PCR Master Mix (Life technologies Grand Island, NY). The amplification protocol consisted of an initial step of 2 min at 50°C and 10 min at 95°C, followed by 45 cycles of 10 s at 95°C and 30 s at 60°C. All experiments were carried out in a ABI PRISM 7000 (Life technologies Grand Island, NY). For each sample, the Ct (Cycle threshold) was used to determine the relative amount of target gene; each measurement was made in triplicate, and normalized to the reference gene *b*-*Glu*, which was also measured in triplicate.

A target gene fold-change (FC) value was finally calculated for each sample using the ΔΔCp method (ΔCp_target-reference sample_ - ΔCp_target-reference calibrator_) using cat ovarian cDNA as calibrator.

### Statistical analysis

Statistical analysis of IHC and molecular expression profiles and correlation with histological parameters and follow-up data were performed with the SPSS advanced statistical package 13.0 (SPSS Inc., Chicago, Illinois). The Spearman correlation test, the Kruskal-Wallis test, the Mann Whitney test, the Wilcoxon test, the Wilcoxon test for paired samples and the sign test were performed. The level of significance was fixed at P < 0.05.

## Results

### Clinical data and histopathology

Mean age of the female cats (n = 81) was 11.6 years (range 1 to 18 years). No significant differences were found between the mean age of animals carrying benign (11.5 years) and malignant neoplasms (12 years). Domestic short hair cats (77.7%) were the most common breed and only 4 Siamese cats were present with a mean age of 14 years. Forty-seven cats were ovariectomized but unfortunately the age at ovariectomy was known only for 10 cats (mean age 7.8 years, range 1 to 12 years, only one cat ovariectomized at 1 year of age).

Histopathology was performed on 156 lesions: 77 malignant lesions (93% of the tumors) (68 primary mammary malignant tumors and 9 lymph node metastases), 6 benign tumors (7% of the tumors), and 73 hyperplastic/dysplastic lesions. Among the malignant tumors, the tubular (22/68, 32%) and the tubulopapillary (18/68, 26.5%) carcinomas were the most common. In addition, 10/68 comedocarcinomas (15%), 6/68 ductal carcinomas (9%), 4/68 solid carcinomas (6%), 3/68 squamous cell carcinomas (4.5%), 2/68 cribriform carcinomas (3%), 2/68 intraductal papillary carcinomas (3%), and 1/68 carcinoma *in*-*situ* were observed. Using the modified Elston and Ellis [32] grading system 20 carcinomas were grade I, 22 were grade II, and 26 were grade III. All “ductal-associated” carcinomas were grade I. Seventeen grade III (65%) mammary carcinomas were associated with peritumoral lymphatic invasion. Benign tumors were two ductal adenomas, two fibroadenomas, and two intraductal papillary adenomas. The most frequently diagnosed hyperplastic/dysplastic lesions were lobular hyperplasia (27/73, 37%), duct ectasia (27/73, 27%), and duct hyperplasia (19/73, 26%). Normal mammary parenchyma was present at the periphery of 8 lesions.

Detailed histopatological evaluation of the RNA Later-preserved samples was not possible but histopathology confirmed the presence of a highly cellular cohesive population of cells in all the 37 cases diagnosed as malignant. More regular and loosely arranged lobular/ductal structures were identified in the 27 samples of the presumptive normal mammary glands.

### IHC evaluations

All 156 lesions and the normal tissues showed 100% positive staining to panCK of the epithelial cells (except one metastasis with 37% panCK + cells). CALP and aSMA were never detected in the luminal epithelial cells. The other IHC results are summarized in Table 2.

**Table 2:** **Expression of markers in luminal neoplastic cells examined by immunohistochemistry**

	**No.**	**Grading**	**PR**	**ERα**	**CK8_** **18**	**VIM**	**CK5**_**6**	**CK14**
			**Percentages of + luminal epithelial cells: Mean ± SD (number of positive samples, range of positive cells)**					
**Normal yglands**	8		24 ± 24.60 (5, 25–72)	38 ± 29 (6, 22–70)	100 ± 0 (8)	100 ± 0 (8) (ductal)	-	100 ± 0 (8) (terminal intralobular ducts)
**Hyperpasia/** **dysplasia**	73		5.59 ± 17 (13, 2.10-87)	10.58 ± 25.54 (25, 1.30-100)	100 ± 0 (73)	100 ± 0 (72)^c^ (ductal)	-	100 ± 0 (29)^e^ (terminal intralobular ducts)
**Benign tumors**	6		15.55 ± 17.47 (3, 27–38)	26.97 ± 38.23 (4, 6–100)	100 ± 0 (6)	42.58 ± 36.62 (5, 7.5-100)	-	-
**Malignant tumors**	68		1.54 ± 9.18 (3, 4.80-70)	2.85 ± 13.86 (6, 2–87.3)	83.45 ± 30.22 (65, 2–100)	53.19 ± 34.73 (62, 0.5-100)	7.76 ± 17.83 (29, 0.5-100)	32.05 ± 32.96 (62, 0.5-100)
DC/IDPC	8	I	(1, 4.80%)^a^	12.38 ± 25.42 (4, 2–73)	100 ± 0 (8)	27.31 ± 28.48 (5, 17.8-80)	-	(1, 34%)^f^
Other subtypes	12	I	(1, 70%)^b^	(1, 7%)^b^	72.67 ± 32.35 (11, 33–100)	51.41 ± 37.74 (11, 1.2-100)	5.69 ± 16.83 (6, 5–56)	32.10 ± 36.62 (9, 0.5-100)
	22	II	-	-	87.18 ± 25.39 (22, 5–100)	57.85 ± 32.88 (21, 7–98)	6.98 ± 10.06 (5, 0.5-28)	37.91 ± 29.96 (22, 0.5-100)
	26	III	(1, 29.70%)^b^	(1, 87.30%)^b^	78.96 ± 35.79 (24, 2–100)	58.04 ± 34.80 (25, 3.3-100)	12.37 ± 25.39 (17, 0.5-100)	35.49 ± 33.84 (23, 0.5-100)
LN Metastases	9		-	-	81.33 ± 35.62 (9, 2–100)	50.83 ± 42.42 (9, 0.5-98)	5.33 ± 12.16 (3, 0.5-32.7)	28.47 ± 35.47 (9, 0.5-97)

SD, standard deviation; ER, estrogen receptor; PR, progesterone receptor; DC, ductal carcinoma; IDPC, intraductal papillary carcinoma; LN, lymph node; ^a^ One case of DC; ^b^One different case of tubulopapilary carcinoma each; ^c^One negative case of epitheliosis; ^e^Exclusively intralobular ducts in hyperplastic lobules were positive; ^f^one case of DC.

Of the hyperplastic/dysplastic tissues 28 cases were ERa-/PR-; only one specimen had normal non-hyperplastic associated mammary tissue that was also ERa-/PR-. Twenty-five out of twenty-eight cases were adjacent to HRs negative tumors and 3/28 cases were not associated with a tumor. There was no association between HRs- status and ovariectomy.

**Figure 1:**
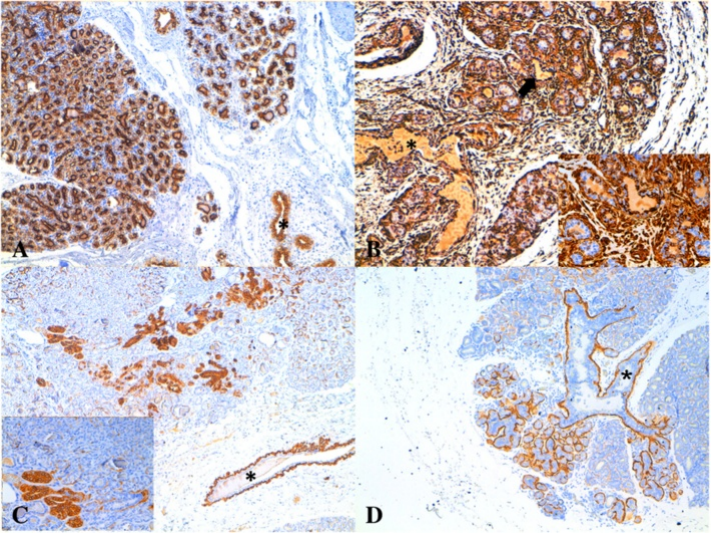
**Hyperplastic mammary gland,** **feline.** IHC, DAB chromogen, hematoxylin counterstain, 5x; **A)** diffuse expression of CK8_18 in luminal cells of lobules and ducts (*); **B)** vimentin expression in the luminal compartment of both interlobular (*) and intralobular ducts (arrow and 40x inset); **C)** CK14 staining observed in luminal cells exclusively in the terminal portion of the intralobular ducts (40x inset) and basal location of the interlobular (*) and intralobular ducts; **D)** CK5_6 positivity detected at basal location of the interlobular (*) and intralobular ducts.

Both in normal and hyperplastic/dysplastic tissues CK8_18 was diffusely expressed in luminal cells (with the exception of the single case of epitheliosis) (Figure 1a). In all cases, VIM was co-expressed with CK8_18 in the luminal compartment of both interlobular and intralobular ducts (Figure 1a,b). In the hyperplastic lobules CK14 was observed in luminal cells exclusively in the terminal portion of the intralobular ducts (Figure 1c). CALP, aSMA, and VIM diffusely stained basal (myoepithelial) cells from the ducts to the lobules, whereas CK14 and CK5_6 were evident at the same basal location, but only in the interlobular and intralobular ducts (Figure 1c,d).

**Figure 2:**
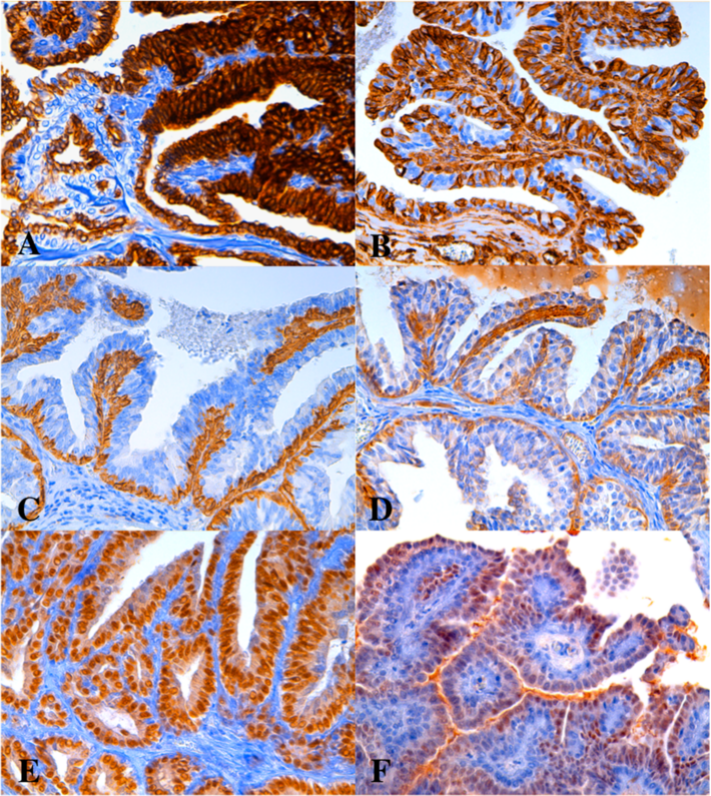
**Mammary gland**, **feline.** IHC, DAB chromogen, hematoxylin counterstain, 10x; **A**-**E)** Intraductal papillary adenoma (“ductal-associated” FMT): **A)** CK8_18- basal/myoepithelial cells and CK8_18+ luminal cells, **B)** VIM + basal/myoepithelial (100%) and luminal (55%) cells, **C)** CK14+ basal/myoepithelial cells, **D)** CK5_6+ basal/myoepithelial cells; **E)** ER expression in luminal cells (100%); **F)** Intraductal papillary carcinoma (“ductal-associated” FMT), ER expression in luminal epithelial cells (70%).

At IHC “ductal-associated” tumors were confirmed by the presence of basal/myoepithelial cells that were CK8_18-/panck + ^low^/CK14+/CK5_6+/CALP+/aSMA+/VIM + (Figure 2a,b,c,d) in association with luminal CK8_18+ cells (Figure 2a) (biphasic tumors) as previously described (Zappulli et al. [28]).

Five out of the six (83%) benign FMTs were positive to HRs (2 ERa+/PR+; 2 ERa+/PR-; 1 ERa-/PR+) (Figure 2e). The ERa-/PR- intraductal papillary adenoma had adjacent non-neoplastic tissue that was also ERa-/PR- and 100% of the luminal neoplastic cells were VIM + in this case. VIM was totally found in 5/6 benign tumors in 42.6% of luminal neoplastic cells, as average (range 7.5-100%) (Figure 2b).

In the FMCs significant differences were found between the “ductal-associated” carcinomas and the “non-ductal-associated” carcinomas. Five out of eight (62.5%) malignant “ductal-associated” tumors were either ERa + (Figure 2f) or PR + in the luminal compartment that showed neither CK5_6 nor CK14 IHC expression. Only one HRs- ductal carcinoma showed luminal CK14 expression (34% cells). Luminal VIM was observed in 5/8 cases (62.5%), they were PR- and either ERa + or ERa-. Similarly to benign tumors and non-neoplastic tissues they were all positive to CK 8_18 in the luminal cells.

**Figure 3:**
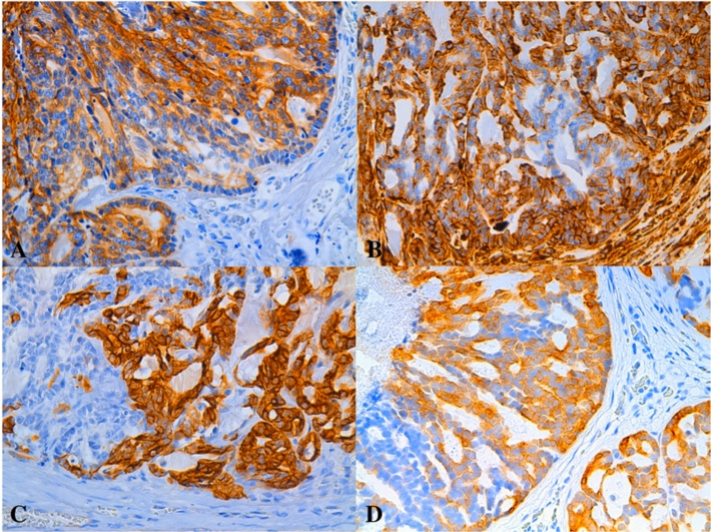
**ERa** **PR-** **simple tubular carcinoma**, **grade II**, **mammary gland**, **feline.** IHC, DAB chromogen0, hematoxylin counterstain, 40x. Expression in neoplastic cells of: **A)** CK8_18 (90%), **B)** VIM (35%), **C)** CK14 (70%), **D)** CK5_6 (28%).

In “non-ductal-associated” FMCs, only 4 cases (6%) were HR positive (7% ER+ + cells, 29.70% PR + cells, 70% PR + cells, 87.3% ER+ + cells, respectively). All these samples were negative for luminal expression of CK5/6. Luminal VIM was present in all (16%, 21%, 80%, and 98% + cells, respectively), and one case was CK14+ (19% luminal cells with 98% VIM+). They all showed 100% CK8_18+ neoplastic luminal cells. In the 94% of the ERa-/PR- FMCs a loss of CK8_18 was found (Figure 3a), whereas there was increased VIM, CK5_6, and CK14 expression (Figure 3b,c,d). 13/29 (45%) and 41/61 (67%) “non-ductal associated” FMCs showed >10% neoplastic CK5_6+ and CK14+ cells, respectively. VIM and CK5_6 expression increased progressively with the grade. The nine metastases showed a pattern of expression similar to ERa-/PR- FMCs for all the markers.

Statistical analyses showed that ERa and PR expression was significantly decreased in FMCs when compared to benign tumors, hyperplastic/dysplastic lesions, and normal tissue, and when compared to paired samples (P < 0.01). CK8/18 was the only other marker that showed a significant decrease in FMCs when compared to benign/non-neoplastic lesions. Positive correlations were found between ERa and both PR and CK8_18 (P < 0.01) and between CK14 and CK5_6 (P < 0.05). A negative correlation was identified between ERa expression and tumor grade (P < 0.01). No significant associations were found with survival and other histopathological parameters.

### mRNA evaluations

In all the examined tissues ERa, ERb, PR and HER2 were all expressed, despite large variances among samples (Table 3). The ERa and PR gene expression were significantly decreased (P < 0.0001 and P < 0.01, respectively) in FMCs compared to matched non-neoplastic mammary tissue. ERβ and HER2 showed no significant difference between tumoral and non-tumoral matched samples. A positive correlation was found exclusively between ERα and PR gene expression both in FMTs (P < 0.001) and in non-neoplastic mammary glands (P < 0.05). Furthermore, ERα expression was negatively correlated with tumor grade (P < 0.05) while for PR the negative correlation was only close to significance (P = 0.06).

**Table 3:** **RT**-**PCR fold**-**change values of markers**

	**Feline mammary carcinomas** **(No. 37)**				**Normal mammary glands** **(No. 27)**			
	**HER2**	**ERα**	**ERβ**	**PR**	**HER2**	**ERα**	**ERβ**	**PR**
**Median value**	1.56	0.12	0.04	0.03	2.16	1.63	0.03	0.31
**Variance**	5.12	2.25	0.01	0.34	7.68	4.33	0.01	3.10
**Range**	0.13-10.1	0.01-8.32	0-0.38	0-3.39	0.59-10.93	0.05-9.94	0-0.43	0-7.40

ER, estrogen receptor alpha; PR, progesterone receptor; HER2, human epidermal growth factor receptor 2.

## Discussion

In this study we present data on the phenotypic and prognostic markers expression in FMTs and associated normal/hyperplastic/dysplastic glands. We found two tumor subgroups: *i*. less aggressive biphasic HRs + (“ductal-associated”) tumors and *ii*. more common and aggressive HRs- heterogeneous carcinomas. We compared proteins levels as detected by IHC with mRNA levels of HRs and we determined ERβ and HER2 expression by RT-PCR in matched samples. We demonstrated the distribution of cell subtypes – both luminal epithelial and basal/myoepithelial lineages – in non-neoplastic glands, and found an unusual and previously undocumented ductal luminal VIM positivity (co-expressed with CK8-18) and a luminal CK14 expression specifically located in the terminal intra-lobular ducts. Both CK14 and CK5_6 staining were increased in HRs- carcinomas.

In our study population (81 queens) the high ratio of malignant (93%) *versus* benign tumors, the frequency of invasive carcinomas of the tubular (28%) and tubulopapillary subtypes (19%), and the relative low frequency of ERα + and/or PR + FMCs correspond with the published data [15,18,33].

In HBC the clinical and pathological classification of subtypes relies on ER, PR, HER2, and Ki-67 IHC labeling and this represents a convenient shorthand substitute, although not identical, to the molecular “intrinsic” subtypes [34,35]. Nevertheless, wide variability of the performance of these tests and inaccurate results (20%) are still detected [6,36,37].

In veterinary medicine, the application of internationally recognized guidelines has not yet been implemented generating an even more imprecise picture of HRs status in FMTs (<40% HRs + FMCs in most studies) [18,21,38]. When assessing the HRs expression by IHC in our feline samples, we detected 12% of normal and 38% of hyperplastic/dysplastic glandular samples that were ER-/PR-; these were always associated with HRs- tumors. Either a technical artifact or a loss of hormone stimulation should be considered as possible explanation for these results. However, data regarding time of samples fixation and age of ovariectomy, that might both affect HRs expression, were not available for our samples. Nevertheless, our data support the idea that aggressive FMCs tend to be HRs- (87%). Also, a progressive loss of HRs expression from non-neoplastic to neoplastic samples as well as from benign to malignant tumors was evidenced in accordance with the literature [18,21,38]. In addition, the subgroup of less aggressive grade I “ductal-associated” carcinomas, defined as ductal and intraductal papillary tumors by morphology and IHC [31], had an increased frequency of ERa positivity when compared to all other carcinomas. Furthermore, ERa and PR expression was positively correlated with CK8_18, a marker of well-differentiated luminal cells, and negatively correlated with tumor grade as previously described [18,21], again indicating of a loss of HRs in less differentiated and more aggressive tumors.

We associated gene expression analysis to the IHC evaluation of ERa and PR for a subset of samples. The significant reduction of ERa and PR proteins in FMCs was confirmed in matched samples, allowing a correction for any subject-related variability.

In our work, we also studied *ERb* expression as already done in HBC [39]. Similarly to HBC, in our feline samples, the *ERb* gene resulted expressed at very low levels in all samples.

Data concerning HER2 status in FMTs are highly controversial with values ranging from 5.5% to 90% of positive tumors probably due to different protocols and evaluation methods [17,19,20,22,23,38,40]. In our work we evaluated HER2 by RT-PCR and we found no significant difference in HER-2 expression between FMCs and matched non-neoplastic tissues. A more than 2-FC increased was detected in 32% FMCs. Only one study analyzed the expression of HER2 mRNA in FMCs that was 3 to 18-FC increased in 6/11 tumors [19]. A previous work from our group analyzed the IHC expression of HER2, Ki-67, and p53 on the same FMTs samples used in this study [22]. When we tested HER2 expression by IHC and strictly applied the Food and Drug Administration (FDA)-approved HercepTest scoring system (DAKO, Glostrup, Denmark) [41], only a single carcinoma scored 3+ [22]. This carcinoma demonstrated the highest RT-PCR relative quantification of HER2 (single case with a 10-FC increase). Other authors tested HER2 IHC expression in FMTs, however variability of methods of assessment makes comparison non-robust [17,19,23,41]. Our data suggests that when strict criteria for HER2 assessment (3+) are used, there are a few cases of HER2-overexpressing FMTs.

On the basis of these and previous results [22] the majority of our FMTs samples had therefore a “triple-negative” phenotype (1/6 benign and 58/68 (85%) malignant).

In humans, there is no consensus on the stratification of TNBCs due to their complex heterogeneity [9,13]. IHC-based surrogates have been discussed to diagnose the TNBCs classes (BLBCs, claudin-low, and normal breast-like) [13].

In our “triple-negative” FMCs we evidenced the appearance of CK5_6 in the 42.6% of the carcinomas (7.8%, average of positive neoplastic cells) and CK14 in the 91% of the carcinomas (32%, average of positive neoplastic cells), comparable to previous findings [17,25], and possibly suggestive of a BLBC phenotype [17]. However, we were unable to demonstrate a significant p53 accumulation in the same dataset (13% of the FMCs) [22], which should be a characteristic of BLBCs in women [13].

A few studies have found decreased expression of adhesion molecules in FMTs [24,25] suggesting a possible EMT-based aggressive phenotype, which is reported for the claudin-low subclass [13]. In the present work, we found vimentin expression (91% of the FMCs) in the neoplastic cells (53% average) a feature also described in other studies [26,42,43]. Vimentin is considered a mesenchymal marker responsible for cell integrity and resistance against stress [44] and its expression in HBC has been addressed as a feature of the claudin-low phenotype [45-47]. However, its prognostic role in HBC in term of both survival and metastases development is not clear [45-48]. One study hypothesized that vimentin-positive invasive HBCs have a direct myoepithelial histogenesis, or a EMT phenotype, or may derive from breast progenitor cells with bilinear (luminal and myoepithelial) differentiation potential [45].

This information would support a significant similarity between the aggressive FMCs and the claudin-low TNBC class.

**Figure 4:**
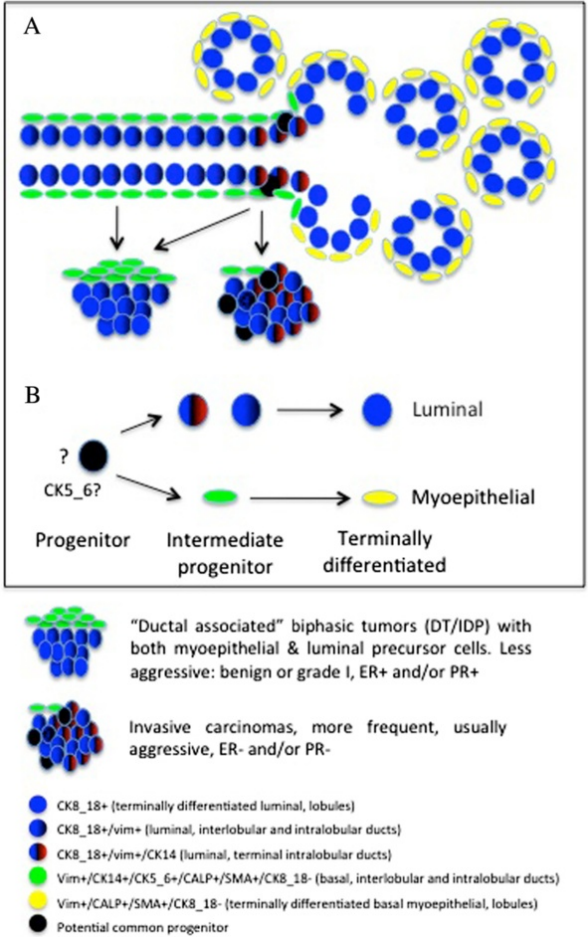
**Potential localization of mammary cell lineages and associated tumour origin. A)** Hypothetical distribution of cell subtypes within the feline mammary gland according to the immunohistochemical analyses for cytoplasmic filaments and associated presumptive cell/site of origin of feline mammary tumors. **B)** Hypothetical lineage differentiation of feline mammary gland cell subtypes. (DT, ductal tumors; IDP, intraductal papillary tumors; ER, estrogen receptor alpha; PR, progesterone receptor; CK, cytokeratin; vim, vimentin; CALP, calponin; SMA, alpha-smooth muscle actin).

Our findings on the cell lineages phenotype and distribution in the non-neoplastic feline mammary gland (see a schematic summary in Figure 4) provide useful insights on the potential site/cell of origin of these aggressive FMCs. We found two separate lineages, both with precursor and terminally differentiated cells that had a different distribution pattern in the non-neoplastic glands. Terminally differentiated luminal (CK8_18+) and myoepithelial (VIM+/CALP+/aSMA+) cells were present within the lobules. The ductal system was characterized by basal intermediate progenitors (VIM+/CK5_6+/CK14+/CALP+/aSMA+) and luminal cells that diffusely co-expressed CK8_18 and VIM. The subgroup of “ductal-associated” tumors overlapped this dual (biphasic) component and had a less aggressive (grade I) HRs + phenotype, suggesting a more stable ductal-differentiation.

Exclusively and consistently at the ductal-lobular junction of the non-neoplastic glands (possibly *terminal end*-*buds* region) the luminal cells stained with CK14. In the more common, aggressive, predominantly triple-negative, “non-ductal associated” FMCs the biphasic nature was not present. A diffuse increase of CK14 and a moderate positivity to CK5_6 were observed.

As indicated by Figure 4, and similarly to what reported for HBCs [3,5], all these data suggest that these “non-ductal associated” FMCs arise at the ductal-lobular junction where potential stem/progenitor cells (CK5_6+) reside to expand the normal glandular lobules and are capable to give rise to CK8_18+/CK14+/VIM + clones responsible for the heterogeneity of these FMCs supporting the idea that tumor arise from stem cells [5,14].

Two hypotheses are instead consistent for the less aggressive “ductal-associated” FMTs. i) They might originate at the same site, but progressing along a more differentiated phenotype with no or minimal stem/progenitor content (CK5_6 negative). ii) Alternatively they might arise in a more proximal ductal region from intermediate progenitor cells (Figure 4).

There is very little data describing the co-expression of cytokeratins (CK8_18) and vimentin in non-tumoral epithelium (*i.e*. human ciliary epithelium and bovine reproductive tract) [49,50]. Usually their co-expression has been associated with drug resistance, invasion and tumor metastasis [48]. In the feline gland the coexpression of cytokeratin and vimentin may indicate a non-terminally differentiated luminal component that is diffusely distributed in the ducts, corroborating the hypothesis that also vimentin positive HBCs (TNBCs) may originate from a precursor cell with bilineage differentiation potential and not from an EMT process [45]. This may also explain why a small subset of the TNBCs are less-aggressive histological subtypes and/or show myoepithelial differentiation (*i.e*. metaplastic and adenoid cystic) [10]. Vimentin has never been described in the non-neoplastic luminal epithelium of the mammary gland of any species, however, it has been described in the so-called ‘cap cells’ of the mice and in the “side-population” of human breast containing the progenitor cell compartment, further validating a potential role of early breast progenitor cells in the pathogenesis of vimentin-expressing breast cancers [45]. A species-specific distribution of these progenitors might explain why in cats mammary cancer is frequently an aggressive, triple-negative, vimentin-positive carcinoma.

## Conclusions

Our study supports the hypothesis that FMCs are generally aggressive HRs negative cancers that manifest an heterogeneous phenotype characterized by basal cytokeratins and vimentin expression. They appear similar to the TNBCs, particularly to the claudin-low subclass and they might originate from progenitor/precursor cells at the ductal-lobular junction. A second subgroup of less common and less aggressive “ductal-associated” FMTs might instead originate from a hierarchically more advanced precursor or from a more distal ductal portion with less stem progenitors. These results indicate that a species-specific phenotype and distribution of cell lineages within the mammary gland might explain the development of species-specific tumor subtypes. The feline species might represent a good model to study a certain type of HBC, to better understand cancer pathogenesis, and to address novel targeted therapies.
